# Case Report: Diagnosis and successful treatment of a rare case of steroid-refractory chronic graft-vs-host disease-related serositis

**DOI:** 10.3389/fimmu.2025.1546599

**Published:** 2025-04-01

**Authors:** Zilin Xiong, Zerong Wang, Xinchuan Chen

**Affiliations:** ^1^ Department of Hematology, West China Hospital, Sichuan University, Chengdu, China; ^2^ West China Clinical Medical College, Sichuan University, Chengdu, China; ^3^ Department of Hematology, The First Affiliated Hospital of Soochow University, Suzhou Medical College of Soochow University, Suzhou, China

**Keywords:** graft-versus-host disease, pleural effusion, pericardial effusion, serositis, hematopoietic stem cell transplantation, Janus kinase inhibitors, baricitinib

## Abstract

Chronic graft-versus-host disease (cGVHD)-related serositis is rare, and primarily presents as polyserous effusions. The low incidence and differential diagnosis impose significant challenges to the prompt diagnosis and the effective treatment of the disease. Here we report the diagnosis and treatment of a 57-year-old woman who experienced recurrent steroid-refractory cGVHD-related serositis after allogeneic hematopoietic stem cell transplantation (allo-HSCT). We thus added baricitinib, a specific JAK1/2 inhibitor, into the treatment regimen for the recurrent disease. The patient’s clinical symptoms and effusions were effectively relieved. Currently, no recurrence of serositis and significant adverse events were observed. The utilization of baricitinib might emerge as a novel promising therapeutic option for the treatment of steroid-refractory GVHD following allo-HSCT.

## 1 Introduction

Chronic graft-versus-host disease (cGVHD) is the major persistent complication following allogeneic hematopoietic stem cell transplantation (allo-HSCT), occurring in 30–70% of transplant patients. The complication and its treatment-related adverse-effects impose significant non-relapse morbidity and late mortality burdens on the allo-HSCT recipients. Serous cavity effusion is a common complication after allo-HSCT but rarely results from cGVHD, with an incidence ranges from 0.99 to 2% (1–3). The 2014 NIH consensus statement highlighted that the clinical presentations of cGVHD-related serositis often co-occur with manifestations of cGVHD in other organs, and should be diagnosed by excluding other potential causes such as drug reactions, infections, relapsing or new malignancies, and other conditions (4). The diagnosis lacks well-accepted criteria for clinical manifestations and recognized characteristic biomarkers. However, decreased albumin levels and increased absolute monocyte counts are frequently observed in this patient population (5). The rarity and complexity of differential diagnosis present substantial obstacles to the early identification and management of cGVHD-related serositis. A limited number of reports suggested a favorable response to glucocorticoids, albeit with a poor prognosis. However, the patient in our case developed steroid-resistance. Then the patient was successfully treated with the addition of baricitinib, a specific Janus-activated kinase (JAK) 1/2 inhibitor, suggesting that baricitinib may be an effective treatment option for the patients who developed steroid-refractory cGVHD-related serositis.

## 2 Case presentation

**Figure 1:**
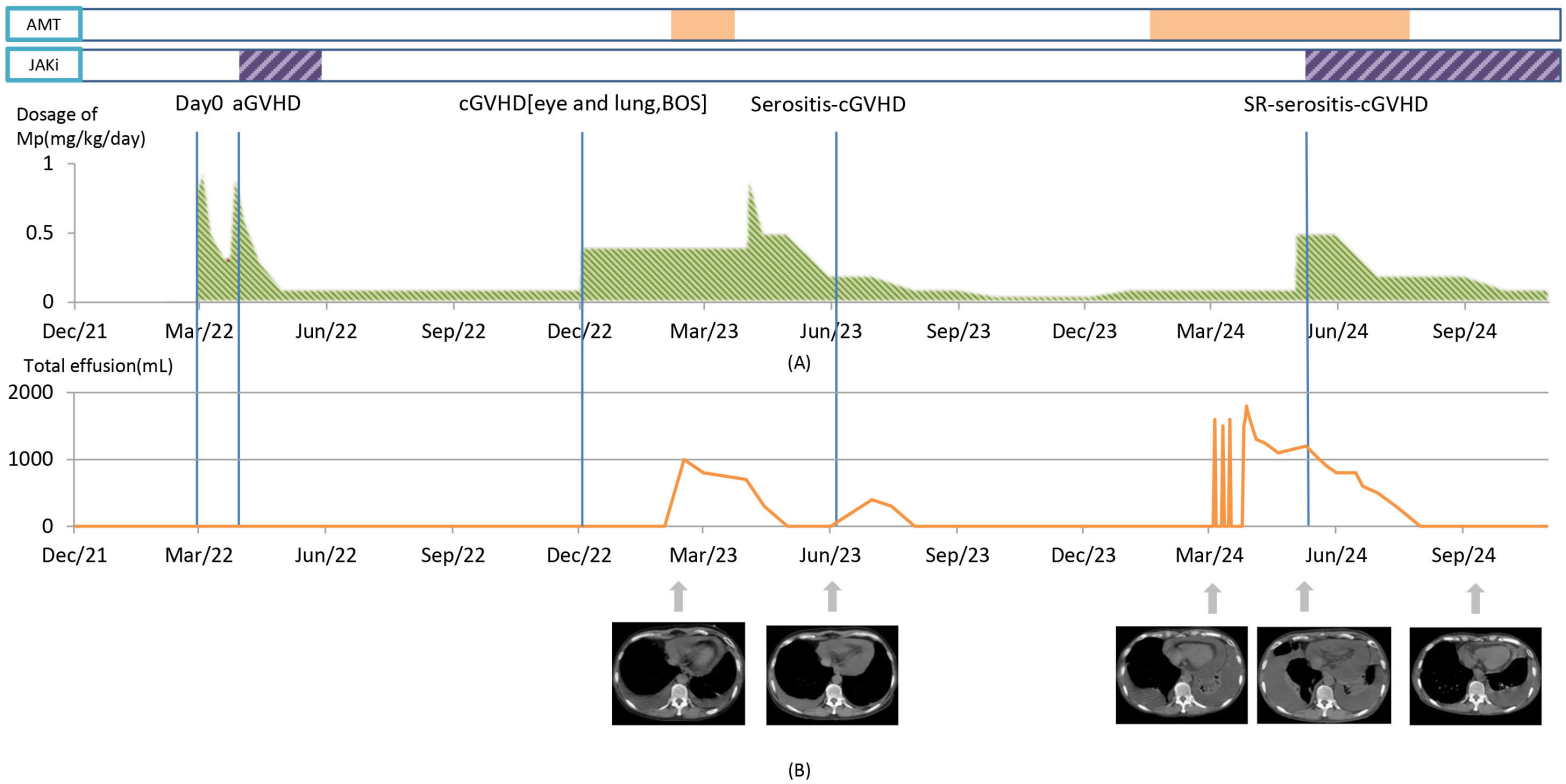
The clinical course, key time points, changes of main therapy, and therapeutic effect. **(A)** Green area: methylprednisolone (MP) dosage changes. Purple area: JAK inhibitors (JAKi) usage (the first time, ruxolitinib; the second time, baricitinib). Orange area: antimicrobial therapy (AMT) usage. Day 0 marks the day of hematopoietic stem cell transplantation. **(B)** The line chart illustrates the relationship between the volume of PLE drainage and the timeline. Below the chart, therapeutic effect is represented by changes in chest CT (mediastinal window) images.

A 57-year-old female patient with myelodysplastic syndrome (MDS-bi_TP53) underwent allo-HSCT. Both the patient and donor were blood type A^+^. The patient received a myeloablative conditioning (MAC) regimen with busulfan, fludarabine and cytarabine, and then a peripheral blood stem cell graft (CD34^+^ cells 2.5×10^6^/kg) from HLA-matched male sibling donor (**Figure 1**). GVHD prophylaxis involved the utilization of reduced doses anti-thymocyte globulin (total dose 2.5mg/kg), post-transplantation cyclophosphamide (total dose 30 mg/kg) and cyclosporine A (CsA). The patient was diagnosed with grade I acute GVHD (aGVHD), according to the modified Glucksberg-Seattle criteria, due to rashes on her chest and back on Day 21. After three days of methylprednisolone (Mp) and CsA, the rash showed progression and was assessed as steroid-resistant aGVHD. Ruxolitinib (a JAK inhibitor) and mycophenolate mofetil (MMF) were administrated. Despite complete remission of aGVHD, the patient developed severe peripheral edema, abdominal distension, and constipation, which were caused by ruxolitinib to lead to discontinuation.

10 months after HSCT, the patient exhibited severe dry eyes and shortness of breath. Ophthalmological examinations identified meibomian gland dysfunction. Spirometry revealed severe obstructive ventilatory dysfunction with the following results: predicted forced vital capacity (FVC%pred), 82.5%; forced expiratory volume in 1 second (FEV1%pred), 41.8%; FEV1/FVC, 0.42; and predicted residual capacity (RV%pred), 154.2% (See **Supplementary Table 1** for the detailed comparison of pulmonary function test before and after transplantation). She was diagnosed with cGVHD [lung cGVHD score 2, with bronchiolitis obliterans syndrome (BOS); eye cGVHD score 2] (according to the National Institutes of Health (NIH) criteria), and was prescribed oral MP at 0.4 mg/kg/day, CsA, azithromycin, imatinib and budesonide and beta-agonist for inhalation, which led to a slight improvement in the above symptoms. One year after allo-HSCT, the patient presented with progressively worsening exertional dyspnea, and no fever, with Karnofsky Performance Status (KPS) score ranging from 30-40. She denied any exposure to specific drugs or poisons and any history of thoracic trauma and pulmonary disease before allo-HSCT. Chest CT revealed mild to moderate amounts of bilateral pleural effusion (PLE) and pericardial effusion. No abnormalities were detected in liver, kidney and cardiac functions based on laboratory tests. Serum albumin levels fluctuated around 35 g/L (reference range: 35~55g/L). C-reactive protein and procalcitonin were not elevated. Cytomegalovirus (CMV) and Epstein-Barr virus (EBV) DNA test were negative. Minimal residual disease (MRD) from bone marrow revealed no signs of tumor relapse. During this hospitalization, PLE was tested multiple times, and the PLE was exudative at some points and transudative at others, according to Light’s criteria. The adenosine deaminase (ADA) was <10 IU/L (ADA > 40 U/L is considered diagnostically significant for tuberculous pleurisy). Multiple cultures, next-generation sequencing (NGS), and exfoliative cytological examinations of effusions were all negative (See **Supplementary Table 2** for further details of examination). The flow cytometry (FCM) analysis of PLE indicated lymphocytes accounted for 87.8% of the number of nuclear cells, mainly T lymphocytes (93.3% of lymphocytes), in which the ratio of CD3+CD4+ and CD3+CD8+ was 1.06, and B cells accounted for 0.1%. Empirical anti-infection treatment with beta-lactamase inhibitors and quinolones was commenced, covering common G+/G- bacteria and acid-fast bacilli. The symptom still showed a progressive trend, with thoracic drainage fluid volume ranging from 400 to 600 ml/day in each side. Given the ineffectiveness of anti-infection treatment and the special FCM result of PLE, MP at 1 mg/kg/day was administrated intravenously. Five days later, the drainage volume decreased to 50-100 ml/day. Eventually, this patient had a mild-to-moderate PLE and remained dyspneic, unable to walk independently for more than 100 meters, with KPS 40-50. However, she no longer required continuous advanced medical support at the medical center or repeated pleuropunctures. Considering the adverse effects of corticosteroids, she was maintained on a low dose of MP at 0.1 mg/kg/day and CsA. The patient’s favorable response to steroid therapy and exclusion of alternative diagnoses led to a retrospective diagnosis of cGVHD-related serositis.

Two years after allo-HSCT, the patient was re-hospitalized due to exacerbated respiratory distress. She had to maintain the semi-Fowler’s position and occasionally even at a high Fowler’s (nearly vertical) position. Chest CT revealed suspected infectious foci with opacities in both lungs, along with moderate to large amounts of bilateral PLE and pericardial effusion. No significant abnormality was observed in organ functions and in results from blood, sputum, and effusion drainage cultures. The drainage was transudate, i.e., no sufficient evidence of infection was indicated. Yet, analysis of the bronchoalveolar lavage (BAL) through next-generation sequencing (NGS) identified Corynebacterium striatum (C. striatum) as the sole pathogen (sequence count: 649,510), for which the targeted therapy with linezolid was given. Sulfamethoxazole/trimethoprim and caspofungin were administrated empirically for Pneumocystis jirovecii pneumonia (PJP) which cannot be rule out in this condition. However, after the antibiotic therapy for seven days, there was no improvement in the symptom of breathless and the volumes of effusions, suggesting alternative etiologies. Considering the recurrent presentations of effusions while the patient had been undergoing long-term steroid therapy, the diagnosis of steroid-refractory cGVHD (SR-cGVHD)-related serositis was made. MP was escalated from to 0.1 mg/kg/day to 0.5 mg/kg/day. She previously benefited from a JAK inhibitor (ruxolitinib) therapy for aGVHD. However, she refused to reuse it because of the severe edema following the previous ruxolitinib treatment. We therefore opted for another JAK inhibitor, baricitinib (2 mg/day), which has not been reported to cause the adverse event of severe edema. With the treatment, the patient’s pericardial and bilateral PLE volumes kept decreasing. About one month after the initiation of baricitinib treatment, MP tapering from 0.5 to 0.2 mg/kg/day was started. Another month later, the drainage tubes were successfully removed, and she was able to seat at low Fowler’s position (15 to 30 degrees). Meanwhile, she was cleared for discharge. About five months after starting baricitinib, the patient could walk independently about 1000 meters, with the physical performance score KPS ranging from 70-80. Consequently, the treatment regimen consisted of a reduced dose of MP (0.1 mg/kg/day) and the maintenance of baricitinib (2mg/day) was commenced. No recurrence of serositis and significant adverse events such as edema and virus infection were observed during 6 months following-up period.

## 3 Discussion

To the best of our knowledge, this is the first report on the successful treatment of steroid-refractory cGVHD-related serositis by the regimen of baricitinib in combination with steroid therapy without causing significant adverse events.

Management of cGVHD is extremely important following allo-HSCT. cGVHD is the leading cause of non-relapse mortality among long-term allo-HSCT survivors. cGVHD-related serositis is a rare disease, thus imposing significant challenges to its prompt diagnosis and effective treatment. It primarily presents as pleural, abdominal, and pericardial effusions, which often lead to hospitalization due to symptoms such as rapid weight gain, edema, and severe dyspnea. These conditions typically respond well to steroid therapy. A retrospective study conducted in 2016 analyzed data from 618 adult patients who allo-HSCT and found that 71 developed PLE. Specifically, 66% of patients developed PLE within the first 100 days post-transplantation, with the majority attributed to infections. Additionally, other causes included implantation syndrome, venous obstruction syndrome (SOS), and congestive heart failure. Only 8 patients were ultimately diagnosed with serositis cGVHD, with the onset of their PLE occurring between 100 days and 5 years after HSCT (6). In a separate cohort study of 281 patients with cGVHD, 12 patients fulfilled the diagnostic criteria for cGVHD-associated serositis. Notably, the effusion in 10 of these 12 patients (71%) exhibited transudate and the mean lymphocyte count was 55.7%, with a range of 7-94% (7).

For instance, Masuda et al. reported in 2024 on an atypical patient who presented with isolated PLE and achieved remission following steroid treatment. this case attributes the massive PLE to cGVHD by excluding other potential causes (such as infection, heart failure, and malignancy) and based on the patient’s history of cGVHD and the rapid response of pleural effusion to corticosteroid therapy (8). An acute infection is often life-threatening for patients with immunodeficiency such as patients undergoing steroid therapy. For the case reported here, the patient had been receiving long-term steroid therapy and presented with a rapid increase in serosal effusion volumes, thus an acute infection was considered as the primary differential diagnosis after excluding other potential causes. Therefore, empirical antimicrobial therapy was initiated promptly. The finding of C. striatum in BAL further complicated the diagnosis. However, given the non-response to antibiotic therapy, and considering her history of sustained long-term steroid therapy and her positive response to baricitinib in combination with escalated doses of MP, we finally concluded the diagnosis as steroid-refractory cGVHD (SR-cGVHD)-related serositis.

Some researchers attempted to use rituximab and extracorporeal photopheresis in addition to systemic steroids, tacrolimus, and sirolimus to treat this rare condition to achieve a good therapeutic result (6). However, intensified immunosuppression is closely linked to an increased risk of infection-related mortality (9). JAK inhibitors such as ruxolitinib have exhibited significant efficacy in treating SR-cGVHD (10). Recent preclinical studies in mouse models have revealed that baricitinib, a more specific JAK1/2 inhibitor, is superior to ruxolitinib in preventing GVHD for at least two reasons: (1) By preserving JAK3-STAT5 signaling, baricitinib is associated with increases in regulatory T cells (Tregs) that suppress GVHD (11), whereas ruxolitinib inhibits JAK3 and Tyk2.9 thus causing other adverse effects; and (2) Baricitinib has a higher half maximal inhibitory concentration (IC50) than ruxolitinib for JAK2, thus JAK2 is not overwhelmingly inhibited by baricitinib compared with ruxolitinib. JAK2 functions both as an early acting hematopoietic growth factor and as a positive regulator of platelet production (12), thus its functions are needed to some degree for the more tolerable and effective treatment of GVHD.

Baricitinib has been approved for the management of rheumatoid arthritis and atopic dermatitis in many countries. Few reports on the clinical use of baricitinib for the treatment of GVHD have been published (13, 14). In the present case of the recurrent SR-cGVHD-related severe serositis, it is an innovative effective treatment transition to use baricitinib instead of ruxolitinib while maintaining a more balanced immune response, thereby optimizing the patient’s overall therapeutic outcome. The use of baricitinib might also lead to shortening in the duration of using high-doses of broadly immunosuppressive agents, which might themselves contribute to morbidity and mortality after allo-HSCT. Our patient’s response to baricitinib is promising, and we anticipate that this case may inform therapeutic strategies for the rare condition.

## 4 Conclusions

In the context of post-allo-HSCT, a case with intractable serosal cavity effusions undergoing steroid therapy was diagnosed with steroid-refractory cGVHD-related serositis, which was successfully treated with baricitinib in combination with methylprednisolone. Baricitinib treatment was well-tolerated and no significant adverse effects were observed in the patient. The utilization of baricitinib, as a specific JAK1/2 inhibitor, may emerge as a novel promising therapeutic option for the treatment of GVHD following allo-HSCT, on which further larger studies are warranted.

## Data Availability

The raw data supporting the conclusions of this article will be made available by the authors, without undue reservation.

## References

[1] Leonard JT Newell LF Meyers G Hayes-Lattin B Gajewski J Heitner S . Chronic GvHD-associated serositis and pericarditis. Bone Marrow Transplant. (2015) 50:1098–104. doi: 10.1038/bmt.2015.105 25961774

[2] Liu YC Gau JP Hong YC Yu YB Hsiao LT Liu JH . Large pericardial effusion as a life-threatening complication after hematopoietic stem cell transplantation-association with chronic GVHD in late-onset adult patients. Ann Hematol. (2012) 91:1953–8. doi: 10.1007/s00277-012-1541-z 22869091

[3] Seber A Khan SP Kersey JH . Unexplained effusions: association with allogeneic bone marrow transplantation and acute or chronic graft-versus-host disease. Bone Marrow Transplant. (1996) 17:207–11. 8640168

[4] Jagasia MH Greinix HT Arora M Williams KM Wolff D Cowen EW . National Institutes of Health consensus development project on criteria for clinical trials in chronic graft-versus-host disease: I. The 2014 diagnosis and staging working group report. Biol Blood Marrow Transplant. (2015) 21:389–401.e1. doi: 10.1016/j.bbmt.2014.12.001 25529383 PMC4329079

[5] Liu YC Chien SH Fan NW Hu MH Gau JP Liu CJ . Risk factors for pericardial effusion in adult patients receiving allogeneic haematopoietic stem cell transplantation. Br J Haematol. (2015) 169:737–45. doi: 10.1111/bjh.2015.169.issue-5 25818840

[6] Modi D Jang H Kim S Deol A Ayash L Bhutani D . Incidence, etiology, and outcome of pleural effusions in allogeneic hematopoietic stem cell transplantation. Am J Hematol. (2016) 91:E341–7. doi: 10.1002/ajh.24435 PMC6852667 27238902

[7] Ballal P Arndt P . A review of chronic graft vs host disease-associated serositis: what we know and don’t. CHEST. (2016) 150:579A. doi: 10.1016/j.chest.2016.08.668

[8] Masuda Y Yamazaki S Honda A Masamoto Y Kurokawa M . Isolated massive pleural effusion as a manifestation of chronic graft versus host disease successfully treated with corticosteroid. Ann Hematol. (2024) 103:1403–7. doi: 10.1007/s00277-024-05643-w PMC10940441 38285080

[9] Sullivan KM Shulman HM Storb R Weiden PL Witherspoon RP McDonald GB . Chronic graft-versus-host disease in 52 patients: adverse natural course and successful treatment with combination immunosuppression. Blood. (1981) 57:267–76. doi: 10.1182/blood.V57.2.267.267 7004534

[10] Zeiser R Polverelli N Ram R Hashmi SK Chakraverty R Middeke JM . Ruxolitinib for glucocorticoid-refractory chronic graft-versus-host disease. N Engl J Med. (2021) 385:228–38. doi: 10.1056/NEJMoa2033122 34260836

[11] Choi J Cooper ML Staser K Ashami K Vij KR Wang B . Baricitinib-induced blockade of interferon gamma receptor and interleukin-6 receptor for the prevention and treatment of graft-versus-host disease. Leukemia. (2018) 32:2483–94. doi: 10.1038/s41375-018-0123-z PMC6168427 29691471

[12] Quintás-Cardama A Vaddi K Liu P Manshouri T Li J Scherle PA . Preclinical characterization of the selective JAK1/2 inhibitor INCB018424: therapeutic implications for the treatment of myeloproliferative neoplasms. Blood. (2010) 115:3109–17. doi: 10.1182/blood-2009-04-214957 PMC3953826 20130243

[13] Holtzman NG Im A Ostojic A Curtis LM Parsons-Wandell L Nashed J . Efficacy and safety of baricitinib in refractory chronic graft-versus-host disease (cGVHD): preliminary analysis results of a phase 1/2 study. Blood. (2020) 136:1. doi: 10.1182/blood-2020-140392 32430499

[14] Shimizu M Shimbo A Takagi M Eguchi K Ishimura M Sugita J . Successful treatment of joint and fascial chronic graft-versus-host disease with baricitinib. Rheumatol (Oxford). (2021) 61:e1–3. doi: 10.1093/rheumatology/keab599 34302457

