# Supplementary material for: Case Report: Diagnosis and successful treatment of a rare case of steroid-refractory chronic graft-vs-host disease-related serositis
Source: Front Immunol. 2025 Apr 1;16:1546599. doi: 10.3389/fimmu.2025.1546599 (PMC11996902; doi:10.3389/fimmu.2025.1546599)
Supplement: Supplementary file 1 [file Table1.docx]

Table 1. Pulmonary function test:

|  | Before HSCT | 1 year after HSCT |
| --- | --- | --- |
| predicted forced vital capacity (FVC%pred) | 137.9% | 82.5% |
| forced expiratory volume in 1 second (FEV1%pred) | 133.1% | 41.8% |
| FEV1/FVC | 0.81 | 0.42 |
| predicted residual capacity (RV%pred) | 113.7% | 154.2% |
