# Supplementary material for: Case Report: Diagnosis and successful treatment of a rare case of steroid-refractory chronic graft-vs-host disease-related serositis
Source: Front Immunol. 2025 Apr 1;16:1546599. doi: 10.3389/fimmu.2025.1546599 (PMC11996902; doi:10.3389/fimmu.2025.1546599)
Supplement: Supplementary file 2 [file Table2.docx]

Table 2.The comparison between the two episodes of progressive PLE in the results of clinical examinations

|  | The first episode | The second episode |
| --- | --- | --- |
| Effusion appearance | Yellow and clear | Yellow and clear |
| Cells counting in effusion (microscopy) | Erythrocytes:  (100-200)×10^6/L | Erythrocytes:  (30-150)×10^6/L |
|  | nucleated cells:  (100-300) ×10^6/L | nucleated cells:  (5-40) ×10^6/L |
|  | Mononuclear cells: 70%-98% | Mononuclear cells:- |
|  | Multinuclear cells:2%-30% | Multinuclear cells:- |
|  | Mesothelial cells:- | Mesothelial cells:- |
| Effusion-Serum total protein gradient | 0.47-0.68 | 0.41-0.55 |
| Effusion-Serum LDH gradient | 0.56-0.97 | 0.32-0.55 |
| the actual value of effusion LDH/ the upper limit of LDH reference in serum | 0.48-0.78 | 0.55-0.60 |
| Effusion glucose/ serum glucose | 1.26-1.45 | 0.6-1.16 |
| BNP in serum (reference: 0-334ng/L) | 140-320 | 140-300 |
| The cardiac EF% by transthoracic echocardiography  (reference: 50-80%) | 78 | 72-77 |
| Serum ALT(reference: 40<IU/L) | 13-30 | 16-26 |
| Serum AST  (reference: 35<IU/L) | 16-36 | 25-38 |
| Serum albumin  (reference:35-55g/L) | 30.3-37.4 | 29.2-41.9 |
| Serum creatinine  (reference: 45-80umol/L) | 66-126 | 54-80 |
| Qualitative of urinary protein | (-) | (-) |
| Pathogenic microorganism culture and staining (including anti-acid staining) | Blood (-) | Blood (-) |
|  | PLE | PLE |
|  | ALF/ sputum | ALF/ sputum |
| Common 17,500 pathogenic microorganism NGS (including bacteria, fungi, viruses and parasites) | blood (-) | blood (-) |
|  | PLE (-) | PLE (-) |
|  | ALF (-) | ALF: C. striatum (sequence count: 649,510) |
| Pathogenic microorganism RT-PCR (including common 13 respiratory viruses) | PLE (-) | PLE (-) |
|  | ALF/ sputum (-) | ALF/ sputum (-) |
| EBV-DNA test  (PCR-fluorescence probe) | Serum (-) | Serum (-) |
| CMV-DNA test  (PCR-fluorescence probe) | Serum (-) | Serum (-) |
| Tuberculosis IGRA | Serum (-) | Serum (-) |
| Xpert and TB-DNA test (PCR- fluorescence probe) | PLE (-) | PLE (-) |
|  | ALF/ sputum (-) | ALF/ sputum (-) |
| Anti-TB antibody | Serum (-) | Serum (-) |
|  | PLE (-) | PLE (-) |
|  | ALF/ sputum (-) | ALF/ sputum (-) |
| The antibody spectrum of anti-ANA, anti-ENA and anti-dsDNA | Serum (-) | Serum (-) |
| MRD FCM | Bone marrow (-) | Bone marrow (-) |
|  | PLE (-) | PLE (-) |

ALF: Alveolar lavage fluid

PLE: pleural effusion
